# Enhancement of Ketone Supplements-Evoked Effect on Absence Epileptic Activity by Co-Administration of Uridine in Wistar Albino Glaxo Rijswijk Rats

**DOI:** 10.3390/nu13010234

**Published:** 2021-01-15

**Authors:** Brigitta Brunner, Enikő Rauch, Csilla Ari, Dominic P. D’Agostino, Zsolt Kovács

**Affiliations:** 1 Department of Biology, ELTE Eötvös Loránd University, Savaria University Centre, Károlyi Gáspár tér 4., 9700 Szombathely, Hungary; brunnerb28@gmail.com (B.B.); raucheniko9810@gmail.com (E.R.); zskovacsneuro@gmail.com (Z.K.); 2 Institute of Biology, Faculty of Sciences, University of Pécs, Ifjúság Str. 6, 7624 Pécs, Hungary; 3 Ketone Technologies LLC., Tampa, FL 33612, USA; ddagosti@usf.edu; 4 Department of Psychology, Behavioral Neuroscience Research Laboratory, University of South Florida, Tampa, FL 33620, USA; 5 Department of Molecular Pharmacology and Physiology, Laboratory of Metabolic Medicine, Morsani College of Medicine, University of South Florida, Tampa, FL 33612, USA; 6 Institute for Human and Machine Cognition, Ocala, FL 34471, USA

**Keywords:** exogenous ketone supplement, uridine, adenosine receptors, absence epilepsy

## Abstract

Both uridine and exogenous ketone supplements decreased the number of spike-wave discharges (SWDs) in a rat model of human absence epilepsy Wistar Albino Glaxo/Rijswijk (WAG/Rij) rats. It has been suggested that alleviating influence of both uridine and ketone supplements on absence epileptic activity may be modulated by A_1_ type adenosine receptors (A_1_Rs). The first aim was to determine whether intraperitoneal (i.p.) administration of a specific A_1_R antagonist 1,3-dipropyl-8-cyclopentylxanthine (DPCPX; 0.2 mg/kg) and a selective adenosine A_2A_ receptor antagonist (7-(2-phenylethyl)-5-amino-2-(2-furyl)-pyrazolo-[4,3-e]-1,2,4-triazolo [1,5-c]pyrimidine) (SCH 58261; 0.5 mg/kg) have a modulatory influence on i.p. 1000 mg/kg uridine-evoked effects on SWD number in WAG/Rij rats. The second aim was to assess efficacy of a sub-effective dose of uridine (i.p. 250 mg/kg) combined with beta-hydroxybutyrate salt + medium chain triglyceride (KSMCT; 2.5 g/kg, gavage) on absence epilepsy. DPCPX completely abolished the i.p. 1000 mg/kg uridine-evoked alleviating effect on SWD number whereas SCH 58261 was ineffective, confirming the A_1_R mechanism. Moreover, the sub-effective dose of uridine markedly enhanced the effect of KSMCT (2.5 g/kg, gavage) on absence epileptic activity. These results demonstrate the anti-epilepsy benefits of co-administrating uridine and exogenous ketone supplements as a means to treat absence epilepsy.

## 1. Introduction

It has been demonstrated that nucleoside levels and metabolic enzymes, transporters, and receptors of nucleosides are unevenly distributed in the central nervous system (CNS) of animals and humans suggesting region-dependent roles of nucleosidergic system in the brain [1]. Indeed, for example, adenosine and uridine have a role in the modulation of physiological and pathophysiological processes in the brain, such as synaptic plasticity, inflammation, and epilepsy [2,3,4]. It was recently demonstrated that uridine has an anti-convulsant and anti-epileptic effect on different animal models [3,5,6] such as a model of human absence epilepsy Wistar Albino Glaxo/Rijswijk (WAG/Rij) rats [7,8], but our knowledge relating to its mechanism of action is far from complete. However, it was suggested that uridine is a signaling molecule in the CNS [9,10,11].

Pyrimidine nucleoside uridine can be synthetized mainly by liver and adipose tissues [12]. As de novo synthesis of pyrimidines in the brain is limited [13] brain utilization is mostly plasma uridine to generate and maintain proper uridine levels for different physiological processes [12]. Uridine molecules can be transported from liver cells to the circulatory system, subsequently enter the brain through the blood–brain barrier and transported through nucleoside transporters into brain cells [14]. Uridine may be metabolized to uridine nucleotides such as uridine triphosphate (UTP) in brain cells [15], which is involved in synthesis of RNA and glycogen molecules as well as membrane lipid phosphatidylcholine [12,16]. Alternatively, uridine may catabolize intracellularly to uracil and subsequently to dihydrouracil by uridine phosphorylase and dihydropyrimidine dehydrogenase, respectively [1,12,15]. Moreover, UTP can be released from brain cells and metabolized extracellularly by ectonucleotidase enzyme cascade to uridine, which uridine can be transported again into the brain cells. The uridine–UTP–uridine cycle can provide local generation of adequate uridine and UTP levels for cellular functions [1,14,15]. In relation to the mechanism of action of uridine in the CNS it was demonstrated, among others, that uridine may bind to its own putative receptor, which receptor has not been cloned yet [11]. It was also suggested that uridine can also modulate different neurotransmitter systems, such as the adenosinergic system, likely via interaction with A_1_ type adenosine receptors (A_1_Rs) [9,11].

A great deal of evidence from retrospective long-term studies, multicenter studies, meta-analysis, randomized clinical controlled trials and Cochrane reviews suggests that ketogenic diets (KDs) are effective in the treatment of different types of epilepsies in children, adolescents, and adults with, for example, drug-resistant epilepsy, (super-refractory) status epilepticus and febrile infection-related epilepsy syndrome [17,18,19,20,21,22]. It has been suggested that not only KDs, but also exogenous ketone supplements (EKSs)- evoked hyperketonemia (increased beta-hydroxybutyrate/βHB and acetoacetate/AcAc) may have therapeutic potential in the treatment of several diseases, such as epilepsy [23,24,25,26]. Ketone bodies can potentiate the repolarization of neuronal membrane, inhibit vesicular glutamate transporters and glutamate release, increase activity of ATP-sensitive potassium channels [27,28,29], enhance the GABAergic and adenosinergic inhibitory effects [27,30,31], and, as a consequence, change electric activities of neurons and brain networks [32], suppressing neuronal (hyper)excitability, and epileptic activity [30,33,34]. Indeed, it was demonstrated that ketosis may have an important role in KD- and EKSs-generated anti-convulsant and anti-epileptic effects [21,25,35,36].

Ketone bodies can cross the blood brain barrier by monocarboxylic transporters, metabolize to acetyl-CoA in the cells and provide a source of energy for the nervous tissue cells via Krebs cycle [23,27]. It is widely accepted that ketone bodies, such as βHB, can exert their alleviating effects on different CNS diseases, such as epilepsy by, for example, modified signaling processes, decreased inflammatory processes, enhanced overall metabolism, and suppressed oxidative stress [37,38,39]. It has also been demonstrated that EKSs, such as ketone ester (KE), and ketone salt (KS), and their mix with medium chain triglyceride (MCT) oils (e.g., KSMCT) generate rapid and sustained nutritional ketosis [25,35,40], which effect decreased not only absence epileptic activity [25], but also the lipopolysaccharide (LPS)-evoked increase in absence epileptic activity in WAG/Rij rats [41]. Moreover, it was also suggested that administration of EKSs may be an alternative metabolic therapy to the KD in the treatment of different CNS diseases, such as epilepsy and psychiatric diseases in which A_1_Rs can evoke modulatory influence [25,42].

One of the most investigated rodent models of human absence epilepsy is the WAG/Rij rat strain [43]. All WAG/Rij rats show bilaterally synchronous and spontaneously occurring spike-wave discharges (SWDs) on electroencephalographic (EEG) recordings, which SWDs are initiated in the hyperexcitable neurons in the somatosensory cortex (cortical focus) [44]. Uridine may modulate absence epileptic activity, for example, through interactions with adenosine receptors [7,45] resulting decrease in neuronal hyperexcitability, and, as a result, in SWD number in WAG/Rij rats. It was suggested that A_2A_ type adenosine receptors (A_2A_Rs) may evoke an increase in absence epileptic activity [46], whereas A_1_Rs may have an alleviating influence on absence epilepsy [4,45]. Exogenous ketone supplements may increase adenosine levels in the CNS and can activate A_1_Rs by which EKSs may modulate pathophysiological processes and diseases in the CNS, such as epilepsy [4,25,31]. These results suggest that adenosine receptors (likely A_1_Rs) could modulate the alleviating effect of not only uridine, but also EKSs on absence epileptic activity. Thus, theoretically, co-administration of uridine and EKSs may enhance their beneficial effects on absence epileptic activity. However, putative modulatory role of inhibitory A_1_Rs or excitatory A_2A_Rs on uridine-evoked beneficial effects on absence epilepsy, as well as influence of co-administration of uridine with EKSs on absence epileptic activity has not been investigated yet. Consequently, first, we tested whether intraperitoneal (i.p.) administration of a non-proepileptic dose of a specific A_1_R antagonist 1,3-dipropyl-8-cyclopentylxanthine (DPCPX) and a non-antiepileptic dose of a selective adenosine A_2A_ receptor antagonist (7-(2-phenylethyl)-5-amino-2-(2-furyl)-pyrazolo-[4,3-e]-1,2,4-triazolo [1,5-c]pyrimidine) (SCH 58261) have modulatory effect on i.p. 1000 mg/kg uridine-evoked alleviating influence on absence epileptic activity in WAG/Rij rats. Moreover, it has been demonstrated previously that KSMCT (2.5 g/kg) gavage-evoked increase in blood βHB level reached the therapeutic level of ketosis (2–7 mmol/L) [24] after one hour of the treatment (~2.3 mmol/L) [47], decreased slightly (≈1.8 mmol/L) about one hour later [25], but did not change significantly the SWD number on the first day of application, compared to control [25]. Theoretically, to increase the effectivity of KSMCT on absence epileptic activity we could increase its dose or use drugs together with KSMCT, which can enhance effect of KSMCT. However, increased dose of EKSs or several drugs may enhance not only their alleviating influences, but also putative adverse effects. Furthermore, administration of compounds—such as uridine—that can enhance the alleviating effects of ketosis could provide relief and flexibility to a broad array of patients struggling with epilepsy (or other disorders), including those on KDs. For example, it would allow the patients to partly liberate their diet and make it more palatable (e.g., reduced % of fat), which may increase efficacy and tolerability and decrease side effects. Consequently, administration of low dose uridine in combination with KDs or exogenous ketone supplements may have strong clinical potential. Thus, to reveal the influence of uridine on KSMCT-generated effect on absence epileptic activity, a smaller and single dose of uridine (i.p. 250 mg/kg) was used in combination with KSMCT (2.5 g/kg, gavage) in the second part of the study. Based on previous studies [4,11,25,41] we hypothesized that adenosine receptor antagonists may modulate the uridine-evoked effect on SWD number and uridine may increase the KSMCT-evoked influence on absence epileptic activity in WAG/Rij rats. The focus of the current study was to test a novel co-administrated therapeutic strategy and mechanism for suppressing absence epileptic activity that is unlike any anti-epilepsy drug (AED) currently in use.

## 2. Materials and Methods

### 2.1. Implantation of Screw Electrodes for Detection of EEG Signals

All parts of experiments were approved similarly to our previous study [25] by the Animal Care and Experimentation Committee of the Savaria University Centre (ELTE, Hungary; license number: VA/ÉBNTF02/85-8/2016). Number of experimental animals for this study was reduced to minimal and pain and suffering were minimized. Male WAG/Rij rats (*n* = 52; 10 months old, 335–357 g) were used (breeding colony of WAG/Rij rats at Savaria University Centre, Szombathely, Hungary). Free access to water and food (Table 1), as well as standard laboratory conditions were provided [25].

Rats were implanted with stainless steel screw electrodes for EEG recording under isoflurane-air mixture (2.0–2.5%) anesthesia [25]. The screw electrodes were placed epidurally over the cortex of the frontal (AP: 2.0 mm; L: 2.1 mm) and parietal (AP: −6.5 mm; L: 2.1 mm) areas [48]. Reference electrodes and ground electrodes were also stainless-steel screw electrodes (implanted above the cerebellar cortex) [49]. All electrodes were soldered to a 10-pin socket and permanently attached to the skull with dentacrylate cement (Ivoclar Vivadent AG, Schaan, Liechtenstein). Lidocaine ointment (5%; EGIS, Budapest, Hungary) was used as post-operative pain relief.

The EEG was recorded in a Faraday cage. EEG signals were fed to an electroencephalograph (NIHON-KOHDEN, Tokyo, Japan)-CED (Cambridge Electronic Design Ltd., Cambridge, UK; POWER 1401 mkII) system. The sampling rate was 500 Hz whereas the bandwidth of the EEG recording was 0.3 Hz to 150 Hz. Both the treatments and handling may induce stress and behavioral changes, which may modify the SWD number [7,50,51]. However, it was observed [7,51] that behavioral changes disappeared within 25–30 min after treatments and normal SWD morphology was also demonstrated. For this reason, data of the first 30 min after the treatments was excluded from the analysis. Thus, the number and time of SWDs were considered between 30 and 270 min of post-treatment time (from 1.00 PM to 5.00 PM). SWDs (a train of asymmetric 7–11 Hz sharp spikes and slow waves) were cut off from the raw data files and were analyzed by FFT analysis [51]. The recording periods were split into 60 min sections and evaluated separately.

### 2.2. Treatment Groups

Based on our previous results on WAG/Rij rats [8,25,46], the effective and well-tolerated dose of uridine (i.p. 250 mg/kg and 1000 mg/kg; Sigma-Aldrich, Inc., Budapest, Hungary), a non-proepileptic dose of DPCPX (i.p. 0.2 mg/kg; Sigma-Aldrich, Inc., Budapest, Hungary), a non-antiepileptic dose of SCH 58261 (i.p. 0.5 mg/kg; Sigma-Aldrich, Inc., Budapest, Hungary) and KSMCT (2.5 g/kg; mix of KS and MCT in a 1:1 ratio; gavage) were used alone and in combination. KS consisted of Na^+^/K^+^-βHB mineral salt [35] whereas MCT oil contained ≈60% caprylic triglyceride and 40% capric triglyceride (Now Foods, Bloomingdale, IL, USA). It has been demonstrated that 1–30% dimethyl sulfoxide (DMSO) solution did not modify absence epileptic activity in WAG/Rij rats [52]. Thus, 10% DMSO (Sigma-Aldrich Inc., Budapest, Hungary) solution was used to dissolve the DPCPX and SCH 58261, whereas uridine was dissolved in saline [8,25,46].

It has been demonstrated previously that effective doses of uridine, DPCPX, SCH 58261, EKSs (such as KSMCT) did not change sleep-waking ratios and averaged time of SWDs (consequently, changes in total time of SWDs were parallel with alteration of SWD number) or discharge frequency within SWDs [7,8,25,41,45]. Consequently, in this study, we focused on changes in SWD number evoked by uridine (250 mg/kg and 1000 mg/kg) alone (group 1, *n* = 6; and group 2, *n* = 6), DPCPX (0.2 mg/kg) alone (group 3, *n* = 6), SCH 58261 (i.p. 0.5 mg/kg) alone (group 4, *n* = 6) and KSMCT alone (group 7, *n* = 8). However, we had no prior data on putative influence of combined administration of DPCPX with uridine, SCH 58261 with uridine, as well as uridine with KSMCT on SWD time. Thus, we investigated effects of these combinations of drugs and KSMCT (group 5, *n* = 6; group 6, *n* = 6; and group 8, *n* = 8) on not only SWD number, but also averaged SWD time and total SWD time (between 150 and 210 min).

**Figure 1:**
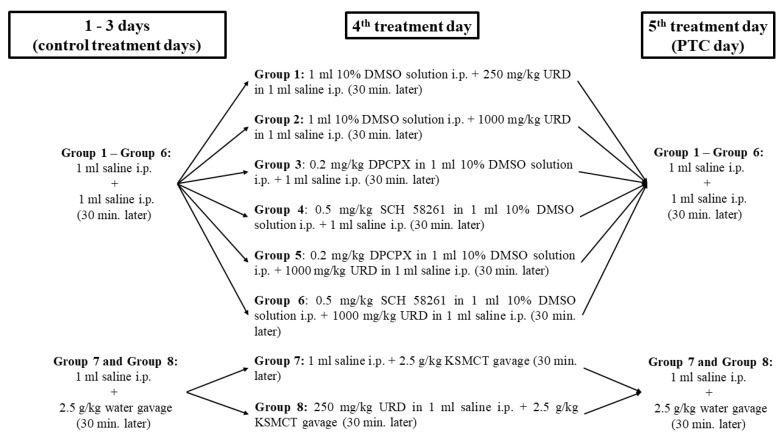
Details of the experimental protocol of the 8 treatment groups. Abbreviations: DMSO, dimethyl sulfoxide; DPCPX, 1,3-dipropyl-8-cyclopentylxanthine; Group: animal group; i.p., intraperitoneal; KSMCT, mix of ketone salt (KS) and medium chain triglyceride (MCT) oil in a 1:1 ratio; PTC day, post-treatment control day; SCH 58261, (7-(2-phenylethyl)-5-amino-2-(2-furyl)-pyrazolo-[4,3-e]-1,2,4-triazolo [1,5-c]pyrimidine); URD, uridine.

Animals were assigned into eight groups (Figure 1). In order to help the rats to adapt to the experimental procedures (e.g., EEG recording), after the two-week recovery period animals were handled daily and were connected to the EEG/CED system for 4 days (once a day, for 4.5 h) (group 1–8). Moreover, on same days, rats of group 7 and group 8 were also gavaged by water (2.5 g/kg/day) for the adaptation of rats to gavage method [25]. To establish averaged control SWD numbers (group 1–8) and control average time and total time of SWDs (group 5, group 6, and group 8), all rats received 1 mL saline i.p. on 3 consecutive days (three control days, first treatment). Thirty min later, animals were injected again with 1 mL saline i.p. (second treatment, group 1–6) or gavaged by 2.5 g/kg water (second treatment, group 7 and 8). After these treatments (similar to fourth and fifth days of experiments), EEG recordings were carried out. On the fourth day of experiments, the rats in group 1 and group 2 were i.p. injected with 1 mL 10% DMSO solution (first treatment) and, 30 min later, with 250 mg/kg, and 1000 mg/kg uridine in 1 mL saline (second treatment), respectively. On the fourth day, animals in group 3 first received 0.2 mg/kg DPCPX alone in 1 mL 10% DMSO solution (first treatment) followed by i.p. 1 mL saline (30 min later; second treatment) (Figure 1). Animals of group 4 were injected by 0.5 mg/kg SCH 58261 in 1 mL 10% DMSO solution (first treatment) and by i.p. 1 mL saline (30 min later; second treatment) on the fourth day. On that day, the animals in group 5 were injected with combined i.p. injection of DPCPX and uridine: 0.2 mg/kg DPCPX in 1 mL 10% DMSO solution (first treatment) and, 30 min later, 1000 mg/kg uridine in 1 mL saline (second treatment) were administered. In the case of group 6, animals were injected similar to group 5 on the fourth day, but the first injection contained 0.5 mg/kg SCH 58261. Animals of group 1–6 were i.p. injected with two saline injections similar to control days on the fifth day (post-treatment control/PTC day) to detect the putative long-lasting effects of treatments on SWD number. Animals of group 7 received i.p. 1 mL saline (first treatment), and 30 min later, gavage of 2.5 g/kg KSMCT was carried out (second treatment) on the fourth day. In relation to group 8, animals were treated similar to group 7 on fourth days, but the first treatment was i.p. 250 mg/kg uridine in 1 mL saline. On the fifth (PTC) day, i.p. administration of 1 mL saline (first treatment) was followed by gavage of 2.5 g/kg water (30 min later; second treatment; group 7 and 8) (Figure 1).

### 2.3. Detection of R-βHB and Glucose Levels

To investigate the effect of KSMCT on blood βHB and glucose levels we measured them on the last (third) control day (control), on the days of the KSMCT gavage (fourth day of experiments) and on the PTC day (fifth day of experiments) after EEG measurements (group 7) by the Precision Xtra™ glucose and ketone monitoring system (Abbott Laboratories, Abbott Park, IL, USA). As the device is able to detect only R-βHB level, the measured βHB level would be lower than the total blood ketone body level (R-βHB + L-βHB + AcAc + acetone). Although the Precision Xtra™ is R-βHB specific, it remains to be determined in a separate, later study whether the use of racemic mixture exogenous ketones is inferior to enantiomerically pure substances (e.g., competition between R and L forms is plausible). Blood, which was used for detection of R-βHB (mmol/L) and glucose (mg/dl) levels, was taken from the tail veins [25,41] (group 7).

### 2.4. Statistical Analysis

Data were presented as means ± standard error of the mean (S.E.M.). The pretreatment control values of SWD numbers (group 1–8) and SWD time (group 5, group 6, and group 8) were the grand average of SWD numbers and time recorded on the three control days. In case of blood level of R-βHB and glucose after KSMCT gavage (group 7), the changes were calculated from the values measured on the last (third) control days. Data analysis was performed by GraphPad PRISM version 6.0a. Analysis was performed using two-way analysis of variance (ANOVA) in order to test the significance of the effect of treatment and time after administration. Significance was calculated by Tukey’s multiple comparisons test [40]. Results were considered significant when *p* < 0.05.

## 3. Results

### 3.1. Effect of Uridine, DPCPX, and SCH 58261 Alone on SWD Number

**Figure 2:**
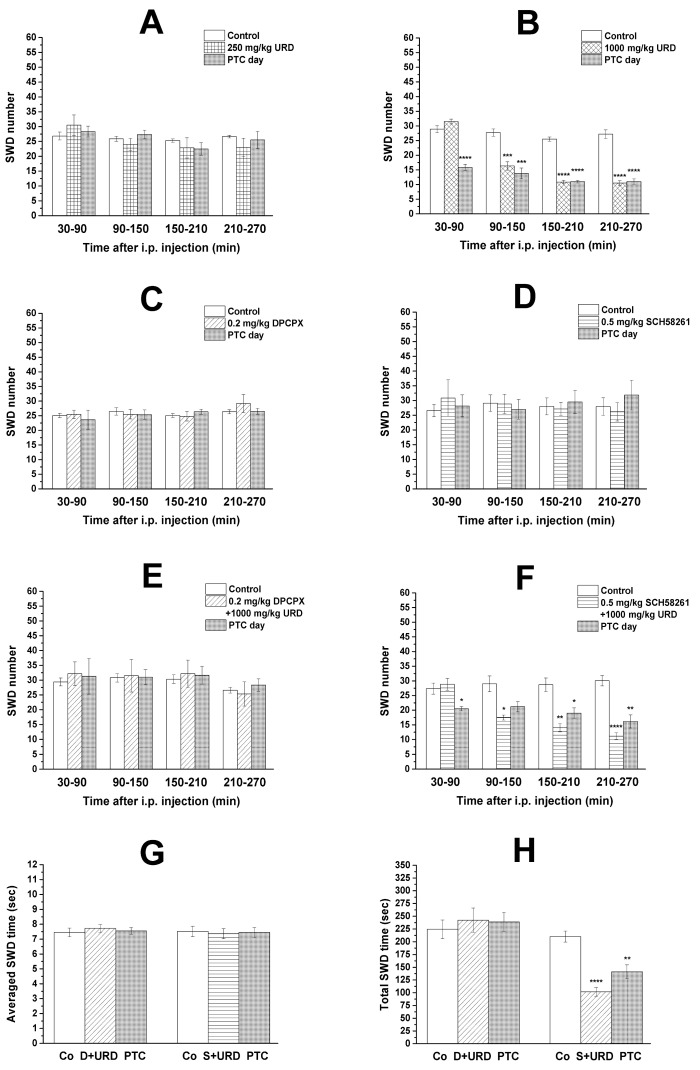
Effect of uridine (i.p. 250 mg/kg; (**A**); i.p. 1000 mg/kg; (**B**)) alone, DPCPX (i.p. 0.2 mg/kg) (**C**) and SCH 58261 (i.p. 0.5 mg/kg) (**D**) alone as well as combined administration of DPCPX (i.p. 0.2 mg/kg) with uridine (i.p. 1000 mg/kg) (**E**) and SCH 58261 (i.p. 0.5 mg/kg) with uridine (i.p. 1000 mg/kg) (**F**) on SWD number. Influence of combined administration of DPCPX (i.p. 0.2 mg/kg) with uridine (i.p. 1000 mg/kg) and SCH 58261 (i.p. 0.5 mg/kg) with uridine (i.p. 1000 mg/kg) on averaged SWD time (**G**) and total SWD time (**H**) between 150 and 210 min. Abbreviations: Co, control; DPCPX, 1,3-dipropyl-8-cyclopentylxanthine; D + Urd: DPCPX + URD; i.p., intraperitoneal; PTC or PTC day, post-treatment control day; SCH 58261, (7-(2-phenylethyl)-5-amino-2-(2-furyl)-pyrazolo-[4,3-e]-1,2,4-triazolo [1,5-c]pyrimidine); S + Urd: SCH 58261 + URD; SWD, spike-wave discharge; URD, uridine. *: *p* < 0.05, **: *p* < 0.01, ***: *p* < 0.001, ****: *p* < 0.0001.

Similar to our previous studies [8,46], normal behavior and typical SWDs were detected in all animals 30 min after the connection of rats to the EEG/CED system. After i.p. 250 mg/kg uridine alone, a trend of non-significant decrease in SWD number was observed during second, third and fourth hours of recording periods, compared to control (group 1, Figure 2A). On the PTC day, significant change in SWD number was not observed (group 1, Figure 2A). We confirmed our previous result [7,8], that i.p. 1000 mg/kg uridine alone significantly decreased the SWD number between 90 and 270 min, compared to control (between 90 and 150 min, *p* = 0.0004; 150 and 210 min as well as 210 and 270 min, *p* < 0.0001) (group 2, Figure 2B). Moreover, significantly decreased SWD number was observed not only on the day of i.p. uridine (1000 mg/kg) injection, but also one day after the uridine administration (PTC day), between 30 and 270 min, compared to control (between 30 and 90 min, 150 and 210 min, as well as 210 and 270 min, *p* < 0.0001; 90 and 150 min, *p* = 0.0004) (group 2, Figure 2B).

We also confirmed our previous result in WAG/Rij rats that i.p. 0.2 mg/kg DPCPX did not evoke proepileptic effect in WAG/Rij rats, not only between 30 and 150 min after i.p. injection of DPCPX (as it was demonstrated previously) [25], but also between 150 and 270 min of recording periods, compared to control (group 3, Figure 2C). Administration of SCH 58261 (i.p. 0.5 mg/kg) did not generate anti-epileptic effect during the four hours recording period (group 4, Figure 2D) and, similarly to DPCPX, it did not change SWD number on PTC days, compared to control (group 3 and group 4, Figure 2C,D).

### 3.2. Effect of Combined Administration of DPCPX and SCH 58261 on Uridine-Evoked Decrease in SWD Number and SWD Time

Combined administration of i.p. DPCPX (0.2 mg/kg) with i.p. uridine (1000 mg/kg) abrogated the anti-epileptic (SWD number decreasing) effect of uridine (1000 mg/kg) alone (group 5; Figure 2E) (between 30 and 90 min, *p* = 0.7946; 90 and 150 min, *p* = 0.9909; 150 and 210 min, *p* = 0.9240; 210 and 270 min, *p* = 0.9514). In addition, i.p. injection of 0.2 mg/kg DPCPX also abolished the uridine (i.p. 1000 mg/kg) injection-evoked decrease in SWD number on PTC day (group 5; Figure 2E) (between 30 and 90 min, *p* = 0.9481; 90 and 150 min, *p* = 0.9964; 150 and 210 min, *p* = 0.9153; 210 and 270 min, *p* = 0.7398). Nevertheless, i.p. administration of SCH 58261 (0.5 mg/kg) mitigated, but did not abolish the alleviating effect of i.p. uridine (1000 mg/kg) on SWD number on days of both uridine injection days (between 90 and 150 min, *p* = 0.0152; 150 and 210 min, *p* = 0.0014; 210 and 270 min, *p* < 0.0001) and on PTC days (between 30 and 90 min, *p* < 0.0311; 90 and 150 min, *p* = 0.0873; 150 and 210 min, *p* = 0.0206; 210 and 270 min, *p* = 0.0019), compared to control (group 6; Figure 2F).

Injection of both DPCPX and SCH 58261 in combination with uridine did not change averaged time of SWDs (group 5, DPCPX + uridine: control vs. treatment day, *p* < 0.7524 and control vs. PTC day, *p* < 0.9601; group 6, SCH 58261 + uridine: control vs. treatment day, *p* < 0.9471 and control vs. PTC day, *p* < 0.9893) (Figure 2G). Consequently, alterations of total time of SWDs were parallel with the change in SWD number (group 5, DPCPX + uridine: control vs. treatment day, *p* < 0.8129 and control vs. PTC day, *p* < 0.8716; group 6, SCH 58261 + uridine: control vs. treatment day, *p* < 0.0001 and control vs. PTC day, *p* < 0.0014) (Figure 2H).

### 3.3. KSMCT-Evoked Changes in Blood R-βHB and Glucose Levels and SWD Number

**Figure 3:**
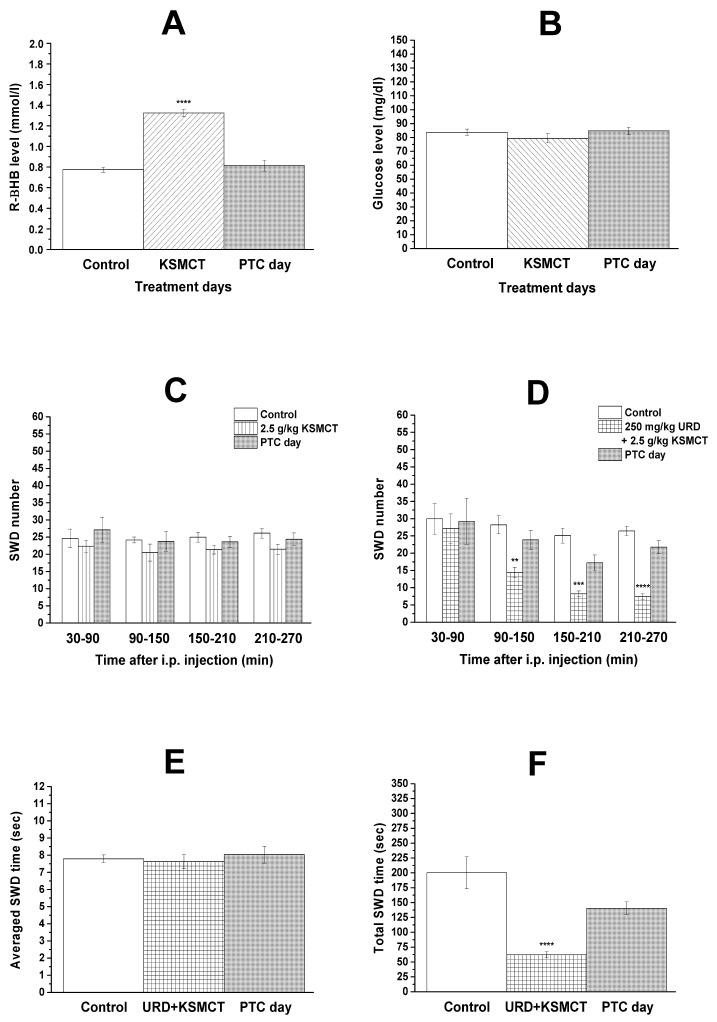
Effect of KSMCT (2.5 g/kg, gavage) alone on blood R-βHB (**A**) and glucose levels (**B**) as well as SWD number (**C**). Influence of combined administration of uridine (i.p. 250 mg/kg) with KSMCT (2.5 g/kg, gavage) on SWD number (**D**) between 30 and 270 min and averaged SWD time (**E**) and total SWD time (**F**) between 150 and 210 min. Abbreviations: i.p., intraperitoneal; KSMCT, mix of ketone salt (KS) and medium chain triglyceride (MCT) oil in a 1:1 ratio; PTC day, post-treatment control day; R-βHB, R-beta-hydroxybutyrate; SWD, spike-wave discharge; URD, uridine. **: *p* < 0.01, ***: *p* < 0.001, ****: *p* < 0.0001.

Gavage of 2.5 g/kg KSMCT significantly increased the blood level of R-βHB on the day of administration (group 7; Figure 3A; *p* < 0.0001) whereas after one day of gavage (on PTC day), R-βHB level returned to the control level (group 7; Figure 3A; *p* = 0.78), compared to control. KSMCT gavage did not change blood level of glucose (group 7; Figure 3B).

We confirmed that single administration of 2.5 g/kg KSMCT (gavage) did not change significantly the SWD number in WAG/Rij rats, not only between 30 and 150 min after gavage [25] but also between 150 and 270 min of recording period, compared to control (group 7, Figure 3C). However, a trend of a non-significant decrease in SWD number was observed during four hours of recording periods, compared to control (group 7, Figure 3C). Moreover, SWD number returned to the control level on the PTC days (group 7, Figure 3C).

### 3.4. Effect of Combined Administration of Uridine and KSMCT on SWD Number and SWD Time

Co-administration of previously established sub-effective dose of uridine (i.p. 250 mg/kg) and KSMCT (2.5 g/kg, gavage) significantly decreased the SWD number during second (*p* = 0.002), third (*p* = 0.0001), and fourth (*p* < 0.0001) hours of recording periods, compared to control (group 8, Figure 3D), suggesting a synergistic effect. Moreover, SWD number decreased between 90 and 270 min of recording period on PTC days, compared to control, but these changes were not significant (between 90 and 150 min, *p* = 0.4969; 150 and 210 min, *p* = 0.0592; 210 and 270 min, *p* = 0.1374).

Combined administration of i.p. 250 mg/kg uridine with 2.5 g/kg KSMCT (group 8) did not change average time of SWDs on the treatment days and PTC days (control vs. treatment day, *p* = 0.9601 and control vs. PTC day, *p* = 0.9033) (Figure 3E); thus, changes in total time of SWDs were parallel with the alteration of SWD number (control vs. treatment day, *p* < 0.0001 and control vs. PTC day, *p* < 0.0535) (Figure 3F).

## 4. Discussion

We confirmed our previous results that i.p. 1000 mg/kg uridine decreased the SWD number between 90 and 270 min of recording period [7,8] (Figure 2B), i.p. 0.2 mg/kg DPCPX did not generate changes in SWD number [25] (Figure 2C) and single administration of 2.5 g/kg KSMCT (gavage) was not able to significantly decrease the SWD number [25] (Figure 3C) in WAG/Rij rats. The present study demonstrated that the A_1_R antagonist DPCPX (i.p. 0.2 mg/kg) abolished the uridine-generated alleviating effects on SWD number, not only on the day of uridine injection (i.p. 1000 mg/kg), but also one day after the injection (PTC day) (Figure 2E). Moreover, a lower (previously established sub-effective) dose of uridine (i.p. 250 mg/kg) alone evoked only a trend of non-significant decrease in SWD number (Figure 2A), but administration of this dose of uridine with KSMCT (2.5 g/kg, gavage) significantly decreased the SWD number (Figure 3D). Finally, we extended our previous results on the effect of both DPCPX and KSMCT on SWD number: i.p. 0.2 mg/kg DPCPX had no proepileptic effects, not only between 30 and 150 min [25], but also during the third and fourth hours of recording period (Figure 2C) and after KSMCT (2.5 g/kg) gavage the trend of non-significant decrease in SWD number was detected longer (between 30–270 min) (Figure 3C) than it was demonstrated previously (30–150 min) [25].

It has been demonstrated that uridine evoked anti-epileptic and anti-epileptogenic effects in different models [3,6], in children with epileptic encephalopathy [53] and in WAG/Rij rats [7,8]. Theoretically, as the uridine is an endogenous molecule, administration of proper doses of uridine in the treatment of epilepsy may be a safe way to evoke anti-epileptic effects without or minimal side effects and considerable risks, compared to pharmacological treatments. Indeed, it has been demonstrated that uridine is a well-tolerated drug with only minor toxic potential, suggesting that uridine and its analogues may be effective and safe anti-epileptic drugs in the treatment of different types of epilepsies [3,4,53,54].

It was suggested that increased level of ketone bodies (e.g., βHB; ketosis) may have therapeutic potential in the treatment of different type of seizures and epilepsies [25,35,55]; thus, EKSs-evoked ketosis [25,47] (Figure 3A) may have therapeutic potential in the treatment of absence epilepsy. Indeed, sub-chronically administered EKSs can increase βHB levels and may decrease SWD number in WAG/Rij rats by time-, dose-, and administration-dependent manner [25,41]. Similar to uridine, it was demonstrated that EKSs, such as KEs, proper doses of KSs and MCTs as well as their combinations (e.g., KEKS, KSMCT, and KEMCT) are well-tolerated, safe and efficient ketogenic agents with little or no side effects [25,35,40,56].

In relation to the putative mechanism of action, for example, it has been demonstrated that ketone bodies may increase the level of adenosine and GABA in the brain, which are endogenous anti-convulsants (modulator and transmitter) that can decrease epileptic activity via their inhibitory receptors A_1_Rs and GABA_A_ receptors, respectively [25,31,57,58]. Uridine may also exert its effect via partly the same anti-convulsant (adenosinergic and GABAergic) systems via interactions with Ado receptors and/or GABA_A_ receptor and putative uridine receptor [11] or by uridine-evoked increase in GABA levels [59]. Moreover, it has been demonstrated that the adenosine released via nucleoside transporters preferentially bind to A_1_Rs [60] and uridine was active in eliciting purine (adenosine) release by means of nucleoside transporters [61]. It has also been demonstrated that GABAergic and adenosinergic systems can regulate absence epileptic activity in WAG/Rij rats [8,45]. However, excitatory A_2A_Rs and GABA_A_ receptors aggravated the absence epileptic activity [8,45,46] whereas A_1_Rs can modulate (decrease) SWD number in WAG/Rij rats [25,41]. Thus, these results suggest that neither GABA_A_ receptors nor A_2A_Rs have a role in the alleviating effect of both uridine and EKSs on absence epileptic activity, but A_1_Rs can modulate their beneficial, modulatory effects on absence epilepsy. These suggestions were strengthened by this study as i.p. administration of DPCPX abrogated beneficial effect of uridine on SWD number (Figure 2E) whereas injection of SCH 58261 did not abolish alleviating effect of uridine on absence epileptic activity (e.g., on SWD number) (Figure 2F). Moreover, co-administration of i.p. uridine and KSMCT (gavage) enhanced the beneficial effect of each other on SWD number, which synergistic effect generated significant decrease in SWD number (e.g., the uridine via interaction between A_1_Rs and putative uridine receptors whereas KSMCT via βHB-induced increase in adenosine level and A_1_R activation) (Figure 3D). Thus, these results above suggest that effect of both uridine and KSMCT on absence epileptic activity may be modulated (at least partly) by A_1_Rs. According to this hypothesis, it was demonstrated that A_1_Rs can inhibit epileptic activity [1,4,45,58,62], brain areas, such as somatosensory cortex, which may have a role in absence epilepsy genesis contain A_1_Rs [1,44,45,63] and activity (expression) of A_1_Rs was decreased in the somatosensory cortex (cortical focus) in presymptomatic WAG/Rij rats [1,4,45]. By synaptic inhibition, A_1_Rs, among others, may reduce Ca^2+^ influx and inhibits excitatory synaptic transmission (e.g., decreases the release of glutamate) as well as increase the activity of ATP-sensitive potassium channels [4,60,64]. These effects can hyperpolarize neuronal membranes, decrease neuronal activity and, as a consequence, may decrease absence epileptic activity.

It is possible that uridine may evoke long-term effect on SWD number by modulation of synaptic plasticity [7,10,11,59,65,66] but our knowledge is not sufficient at present to explain the exact mechanism(s), by which uridine evokes long-lasting effects on SWD number (on PTC days) (Figure 2B) and by which DPCPX abolished this effect of uridine (Figure 2E). However, all of the results above suggest that long-term co-administration of uridine with KSMCT may enhance their anti-epileptic influence day by day, which treatment would be more effective in the treatment of absence epilepsy than their single administration. However, new studies are needed to demonstrate the putative alleviating effect of chronic co-administration of uridine and KSMCT on absence epileptic activity not only in animals, but also in humans.

## 5. Conclusions

The results suggest that co-administration of a sub-effective dose of uridine with KSMCT in WAG/Rij rats evokes a significant and synergistic effect on suppressing SWD number, which is mediated, in part, by A_1_R signaling. The co-administration strategy of uridine and/or EKSs with different anti-epileptics can promote development of more effective treatments in different types of epilepsies, such childhood absence epilepsy and especially therapy-resistant epilepsies. However, further animal and clinical studies should be conducted to reveal the exact mechanism(s) of action of uridine-, EKSs-, and adenosine-evoked effects (administered alone or in combination) on epileptic activity, and how this may further augment the efficacy of AEDs or other standard of care therapies.

## Figures and Tables

**Table 1:** Macronutrient ratios of rodent standard diet (more details about the ingredients of diet can be found in Supplementary Figure S1).

% Cal from Fat	10.0
% Cal from Protein	23.0
% Cal from Carbohydrates	67.0
kcal/g	3.3

## Data Availability

The data presented in this study are available on request from the corresponding author.

## Supplementary Materials

The following are available online at https://www.mdpi.com/2072-6643/13/1/234/s1, Figure S1: A complete feed for rats and mice: ssniff RM-Z+H diet.

## Abbreviations

A_1_Rs	A_1_ type of Ado receptors
A_2A_Rs	A_2A_ type of Ado receptors
AcAc	acetoacetate
CNS	central nervous system
DMSO	dimethyl sulfoxide
DPCPX	1,3-dipropyl-8-cyclopentylxanthine
EKSs	exogenous ketone supplements
IL-1β	interleukin-1β
i.p.	intraperitoneal
KD	ketogenic diet
KS	ketone salt
KSMCT	mix of ketone salt (KS) and medium chain triglyceride (MCT) oil in a 1:1 ratio
LPS	lipopolysaccharide
MCT	medium chain triglyceride
NF-κB	nuclear factor-κB
PTC day	post-treatment control day
R-βHB	R-beta-hydroxybutyrate
SCH 58261	(7-(2-phenylethyl)-5-amino-2-(2-furyl)-pyrazolo-[4,3-e]-1,2,4-triazolo [1,5-c]pyrimidine)
SWD	spike-wave discharge
WAG/Rij	Wistar Albino Glaxo/Rijswijk
