# Supplementary material for: Enhancement of Ketone Supplements-Evoked Effect on Absence Epileptic Activity by Co-Administration of Uridine in Wistar Albino Glaxo Rijswijk Rats
Source: Nutrients. 2021 Jan 15;13(1):234. doi: 10.3390/nu13010234 (PMC7830695; doi:10.3390/nu13010234)
Supplement: Supplementary file 1 [file nutrients-13-00234-s001.pdf]

Rat/Mouse breeding & maintenance

RM-Z+H, fortified (autoclavable / for γ-irradiation)

Complete feed for rats & mice

ssniff

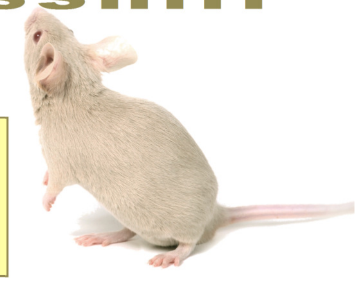

Description

This diet has been designed for breeding and maintenance of rats and mice. It is characterized by medium high protein and energy contents. The diet is vitamin fortified.

The diet is intended for ad libitum feeding. The animals should have free access to fresh water

**Gross Energy (GE)**	**16.3**	**MJ/kg**
**Metabolizable Energy (ME)** [1)]	**13.8**	**MJ/kg**

1) = Physiological fuel value (Atwater); correspond to 3,295 kcal/kg

ME - pig = 13.3 MJ/kg

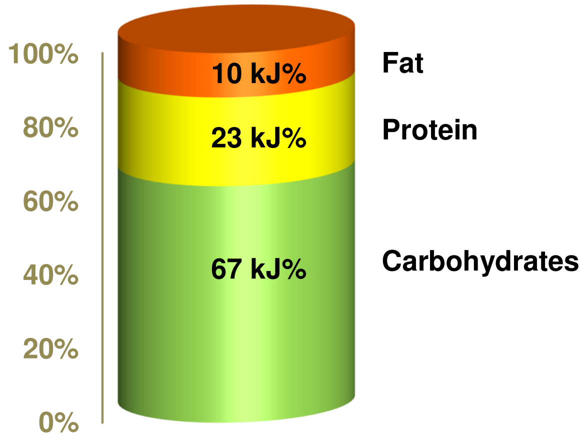

Crude Nutrients	[%]
Crude protein (N x 6.25)	19.2
Crude fat	3.5
Crude fibre	3.8
NDF	14.2
ADF	5.7
Crude ash	6.2
Starch	38.3
Sugar	5.0
N free extracts	55.3

Minerals	[%]
Calcium	1.00
Phosphorus	0.70
Ca / P	1.43 : 1
Sodium	0.22
Magnesium	0.24
Potassium	0.90

Fatty acids	[%]
C 12:0	——
C 14:0	0.01
C 16:0	0.47
C 18:0	0.09
C 20:0	0.01
C 16:1	0.01
C 18:1	0.69
C 18:2	1.92
C 18:3	0.23

Amino acids	[%]
Lysine	1.10
Methionine	0.53
Cystine	0.36
Met+Cys	0.89
Threonine	0.69
Tryptophan	0.24
Arginine	1.14
Histidine	0.49
Valine	0.92
Isoleucine	0.79
Leucine	1.39
Phenylalanine	0.87
Phe+Tyr	1.47
Glycine	0.87
Glutamic acid	4.26
Aspartic acid	1.66
Proline	1.36
Serine	0.98
Alanine	0.87

Vitamins	per	kg
Vitamin A	25,000	IU
Vitamin D_3	1,500	IU
Vitamin E	135	mg
Vitamin K (as MNB)	20	mg
Thiamine (B_1)	86	mg
Riboflavin (B_2)	32	mg
Pyridoxine (B_6)	31	mg
Cobalamin (B_{12})	150	µg
Nicotinic acid	169	mg
Pantothenic acid	62	mg
Folic acid	10	mg
Biotin	740	µg
Choline	2,060	mg

Trace elements	per	kg
Iron	195	mg
Manganese	65	mg
Zinc	93	mg
Copper	15	mg
Iodine	2.1	mg
Selenium	0.4	mg

Dietary composition

Wheat and wheat products, soybean products, barley, corn gluten feed, minerals, vitamins & trace elements, soybean oil, L-lysine HCl, DL-methionine.

Main products		Bag size
S8106-S011	15 mm pellets, autoclavable	25 kg
S5677-S022	10 mm pellets	5 / 10 kg

Production and Sale
ssniff Spezialdiäten GmbH
Phone: +49-(0)2921-9658-0
Fax: +49-(0)2921-9658-40
E-Mail mail@ssniff.de
www.ssniff.de / www.ssniff.com
